# In silico reconstruction of the gene network for cytokine regulation of ASD-associated genes and proteins

**DOI:** 10.18699/vjgb-25-105

**Published:** 2025-12

**Authors:** N.M. Levanova, E.G. Vergunov, A.N. Savostyanov, I.V. Yatsyk, V.A. Ivanisenko

**Affiliations:** Institute of Cytology and Genetics of the Siberian Branch of the Russian Academy of Sciences, Novosibirsk, Russia; Institute of Cytology and Genetics of the Siberian Branch of the Russian Academy of Sciences, Novosibirsk, Russia Scientific Research Institute of Neurosciences and Medicine, Novosibirsk, Russia Novosibirsk State University, Novosibirsk, Russia; Institute of Cytology and Genetics of the Siberian Branch of the Russian Academy of Sciences, Novosibirsk, Russia Scientific Research Institute of Neurosciences and Medicine, Novosibirsk, Russia Novosibirsk State University, Novosibirsk, Russia; Institute of Cytology and Genetics of the Siberian Branch of the Russian Academy of Sciences, Novosibirsk, Russia; Institute of Cytology and Genetics of the Siberian Branch of the Russian Academy of Sciences, Novosibirsk, Russia

**Keywords:** autism spectrum disorder (ASD), neurodevelopmental disorders, cytokines, automatic text analysis of scientific publications, ASD pathogenesis, ASD treatment, computer reconstruction of gene networks, расстройства аутистического спектра (РАС), нарушения нейроразвития, цитокины, автоматический анализ текстов научных публикаций, патогенез РАС, терапия РАС, компьютерная реконструкция генных сетей

## Abstract

Accumulated evidence links dysregulated cytokine signaling to the pathogenesis of autism spectrum disorder (ASD), implicating genes, proteins, and their intermolecular networks. This paper systematizes these findings using bioinformatics analysis and machine learning methods. The primary tool employed in the study was the ANDSystem cognitive platform, developed at the Institute of Cytology and Genetics, which utilizes artificial intelligence techniques for automated knowledge extraction from biomedical databases and scientific publications. Using ANDSystem, we reconstructed a gene network of cytokine-mediated regulation of autism spectrum disorder (ASD)-associated genes and proteins. The analysis identified 110 cytokines that regulate the activity, degradation, and transport of 58 proteins involved in ASD pathogenesis, as well as the expression of 91 ASD-associated genes. Gene Ontology (GO) enrichment analysis revealed statistically significant associations of these genes with biological processes related to the development and function of the central nervous system. Furthermore, topological network analysis and functional significance assessment based on association with ASD-related GO biological processes allowed us to identify 21 cytokines exerting the strongest influence on the regulatory network. Among these, eight cytokines (IL-4, TGF-β1, BMP4, VEGFA, BMP2, IL-10, IFN-γ, TNF-α) had the highest priority, ranking at the top across all employed metrics. Notably, eight of the 21 prioritized cytokines (TNF-α, IL-6, IL-4, VEGFA, IL-2, IL-1β, IFN-γ, IL-17) are known targets of drugs currently used as immunosuppressants and antitumor agents. The pivotal role of these cytokines in ASD pathogenesis provides a rationale for potentially repurposing such inhibitory drugs for the treatment of autism spectrum disorders

## Abstract (ru)

Многочисленные исследования подтверждают связь нарушений цитокиновой регуляции с развитием расстройств аутистического спектра (РАС) на уровне генов, белков и их межмолекулярных взаимодействий. В работе эти данные были систематизированы с применением биоинформатического анализа и методов машинного обучения. Главным инструментом в исследовании являлась когнитивная система ANDSystem, разработанная в Институте цитологии и генетики СО РАН и задействующая методы искусственного интеллекта для автоматического извлечения информации из биомедицинских баз данных и текстов научных публикаций. С использованием ANDSystem была реконструирована ассоциативная генная сеть цитокиновой регуляции генов и белков, ассоциированных с РАС. В результате анализа удалось идентифицировать 110 цитокинов, которые, согласно воссозданной сети, регулируют активность, деградацию и транспорт 58 белков, вовлеченных в развитие РАС, а также экспрессию 91 гена, ассоциированного с этими расстройствами. Анализ перепредстав

## Introduction

DSM-5 (Diagnostic and Statistical Manual of Mental Disorders, Fifth edition) classifies autism spectrum disorder (ASD) as a category of neurodevelopmental conditions exhibiting a substantial genetic component, with diagnosis predicated solely on behavioral criteria (American Psychiatric Association, 2013). The core diagnostic profile of ASD comprises persistent deficits in social communication and reciprocal social interaction, co-occurring with restricted, repetitive patterns of behavior, interests, or activities. Contemporary diagnostic frameworks mandate the manifestation of these symptoms during the early developmental period. While their severity can vary, certain individuals may develop compensatory strategies through learned behaviors, which can mask underlying deficits. A substantial heterogeneity is observed in the behavioral phenotypes associated with ASD (Van der Zee, Derksen, 2021). Furthermore, the neurophysiological features associated with autism were identified not only in diagnosed individuals but also in the general population (Harms et al., 2010; Tsai et al., 2013; Tseng et al., 2015).

ASD classification delineates idiopathic forms, lacking clear genetic correlates, from syndromic forms, which are defined by monogenic mutations and associated comorbid features (Ziats et al., 2021). А considerable subset of syndromic ASD cases is driven by mutations disrupting the mTOR signaling pathway, leading to its persistent hyperactivation (Ganesan et al., 2019). A prior bioinformatic analysis utilizing the SFARI Gene database (Abrahams et al., 2013) demonstrated that approximately 58 % of genes harboring ASD-associated mutations are directly linked to the mTOR signaling pathway (Trifonova et al., 2019). The mTOR protein (mechanistic target of rapamycin) is a serine/threonine kinase that serves as the central component of two protein complexes: mTORC1 and mTORC2. Rapamycin-sensitive mTORC1 responds to nutrient availability and growth factors, regulating cell growth and metabolism. mTORC2, in contrast, is largely rapamycin-insensitive and is activated in response to stress and growth factor signaling, regulating cell survival and proliferation processes (Ragupathi et al., 2024).

mTOR signaling pathway plays a critical regulatory role in diverse physiological processes, including cellular and tumor growth (Onore et al., 2017), immune function (Liu et al., 2015), as well as memory formation and neural circuit plasticity (Hoeffer, Klann, 2010). Furthermore, constitutive hyperactivation of this pathway has been shown to suppress autophagy (McMahon et al., 2012) and impair normal synaptic pruning mechanisms (Tang et al., 2014).

Synaptic pruning is a fundamental neurodevelopmental process involving the microglia-mediated elimination of superfluous synaptic connections persisting from development through adulthood. This refinement mechanism enhances the efficiency of neural transmission and facilitates the reallocation of metabolic and computational resources to behaviorally relevant circuits, thereby underlying effective learning and long-term memory formation (Navlakha et al., 2015). Impairments in this pruning cascade are implicated in the neuropathology of ASD, manifesting as an increase in dendritic spine and synaptic density across both supra- and infragranular layers of the frontal, temporal, and parietal cortices (Hutsler, Zhang, 2010).

Microglia, central to the process of synaptic pruning, are integral to the CNS immune environment, where their activity is modulated by cytokine signalling. Moreover, as a major source of pro-inflammatory cytokines in the brain, microglia function as critical orchestrators of neuroinflammatory processes and possess the capacity to induce or modulate diverse cellular responses (Smith et al., 2012). Postmortem analyses of individuals with ASD have revealed hallmarks of neuroinflammation associated with classical (M1) microglial activation, with documented elevations in interferon IFN-γ and cytokines IL-1β, IL-6, IL-12p40, TNF-α, and CCL2 in both brain tissue and cerebrospinal fluid (Vargas et al., 2005; Li et al., 2009; Morgan et al., 2010).

Cytokines provide regulatory signaling essential for normal early brain development, synaptic plasticity, and the preservation of brain homeostasis. Pronounced alterations in the cytokine milieu disrupt fundamental neurodevelopmental mechanisms such as neuronal migration and differentiation, ultimately leading to the emergence of behavioral deficits (Ashwood et al., 2011). Moreover, comparative analyses of plasma and serum cytokine levels further reveal statistically significant alterations in the immunological profile of individuals with ASD relative to neurotypical controls (Onore et al., 2017). Therefore, a systemic immune regulatory imbalance perpetuates a state of chronic neuroinflammation in ASD.

In this study, we employed artificial intelligence (AI)-based software tools to reconstruct associative gene networks, aiming to identify and systematize regulatory interactions between cytokines and ASD-associated genes and proteins. The analysis was performed using the ANDSystem cognitive platform (Ivanisenko V.A. et al., 2015), a tool specifically designed for automated extraction and integration of data from scientific literature and biological databasesThe objective of this research was to reconstruct and analyze the gene network of cytokine-mediated regulation of ASD-associated genes and proteins, with the specific goal of identifying promising cytokine targets for ASD immunomodulation therapy

Network analysis identified 110 cytokines regulating activity, degradation, and transport of 58 ASD-associated proteins, alongside influencing the expression of 91 ASD-related genes. Gene Ontology enrichment analysis revealed significant involvement of these genes in CNS development and function. Among the 21 cytokines exerting the greatest influence on the network, eight (TNF-α, IL-6, IL-4, VEGFA, IL-2, IL-1β, IFN-γ, IL-17) are targeted by existing immunosuppressive and antitumor drugs. The identified role of these cytokines in ASD pathogenesis provides a strong foundation for exploring drug repurposing strategies targeting them.

## Materials and methods

The study’s first phase involved in silico reconstruction of a network mapping cytokine interactions with ASD-associated proteins and genes (consolidated gene network). To achieve the most comprehensive coverage of these regulatory interactions, five specialized gene subnetworks reflecting different pathways of cytokine influence were first reconstructed (Supplementary Table S1)1. These subnetworks were subsequently integrated into a consolidated gene network.


Supplementary Materials are available in the online version of the paper: https://vavilov.elpub.ru/jour/manager/files/Suppl_Levanova_Engl_29_7.pdf


The second phase comprised a structural bioinformatic analysis of the integrated network and functional annotation of its components using Gene Ontology to identify ASD-relevant biological processes. This was followed by prioritization of cytokines according to their predicted regulatory impact on ASD-associated genes and proteins

The final stage focused on identifying promising targets for immunomodulatory ASD therapy among the cytokines demonstrating the highest significance in the conducted ana- lysis.

**Stage 1.** A set of ASD-associated genes (234 genes) was obtained from the SFARI Gene database (Abrahams et al., 2013) (https://gene.sfari.org). The sample included genes annotated in this database as having a high confidence of association with ASD (Category 1 according to the database’s internal scoring system). Lists of cytokine genes (186 genes) and cytokine receptor genes (114 genes) were compiled using data extracted from the Human Protein Atlas (HPA) (https://www. proteinatlas.org/), a comprehensive knowledge base focused on the spatial localization and expression profiles of human proteins in tissues, cells, and organs (Uhlén et al., 2015).

Gene networks were reconstructed using the ANDVisio software (Demenkov et al., 2012), which utilizes data from the ANDSystem’s knowledge base for network reconstruction and structural analysis. ANDSystem is designed for automated analysis of scientific publications and databases and employs ontological modeling, graph analysis, and natural language processing mechanisms (Ivanisenko V.A. et al., 2019; Ivanisenko T.V. et al., 2020, 2022, 2024).

A consolidated network was assembled from subnetworks reconstructed using ANDVisio’s ʻPathway Wizardʼ. This tool enables the retrieval and visualization of gene networks from the ANDSystem knowledge base that match specified query templates. Five individual subnetworks were initially constructed using five distinct query templates (Table S1) and subsequently merged into a unified graph.

**Stage 2.** Gene Ontology (GO) term enrichment analysis for biological processes (GO_BP) (Ashburner et al., 2000) was performed on the consolidated gene network utilizing the DAVID bioinformatics platform (Huang et al., 2009; Sherman et al., 2022) (https://davidbioinformatics.nih.gov/). DAVID provides functional gene annotation and evaluates the statistical significance of GO term enrichment within gene sets against user-defined confidence thresholds

Network topology analysis and cytokine ranking were performed using the statistical tools implemented in ANDVisio. Cytokines were evaluated based on two centrality metrics: betweenness centrality, defined as the fraction of the shortest paths traversing a node, and degree centrality, representing the number of its direct connections. Both parameters serve as measures of nodal influence within the network, where higher values correspond to greater functional significance. Furthermore, pathway-based prioritization of cytokines was conducted using a custom Python 3.10 script to assess their representation in ASD-associated biological pathways.

**Stage 3. ** Cytokines identified through prior analysis were subsequently evaluated as potential targets for pharmacological intervention. This assessment incorporated data from the DrugBank (Knox et al., 2024) (https://go.drugbank.com/) and GETdb (Zhang et al., 2024) (https://togodb.org/db/getdb) databases

## Results of gene network reconstruction and analysis

**Reconstruction of cytokine interactions with ASD-associated proteins and genes**

**Fig. 1.**
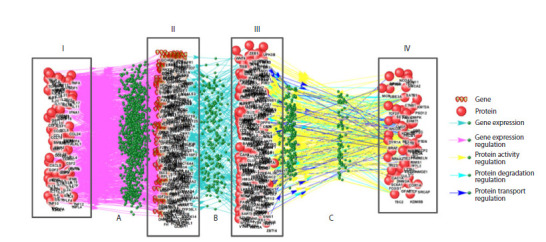
Example of subnetwork reconstruction: modeling cytokine interactions with ASD-associated proteins via Pathway Wizard software using Template 1 from Supplementary Table S1. Roman numerals indicate: I, cytokines regulating ASD-associated proteins and genes, II, mediator genes, III, mediator proteins, IV, ASD-associated proteins regulated by cytokines through signaling pathways. Letters denote: A, gene expression regulation, B, gene expression, C, regulation of protein activity, transport, and degradation

During the initial research phase, five sub-networks were reconstructed using the Pathway Wizard software (Fig. 1). The subnetwork reconstruction utilized two datasets: ASDassociated gene set from the SFARI database (https://gene. sfari.org) and a list of cytokines and their receptors obtained from the Human Protein Atlas database (https://www.proteinatlas. org/).

Following automated reconstruction, all retrieved connections and network elements were manually reviewed against source publication texts to eliminate errors arising from inaccurate information extraction

**Fig. 2.**
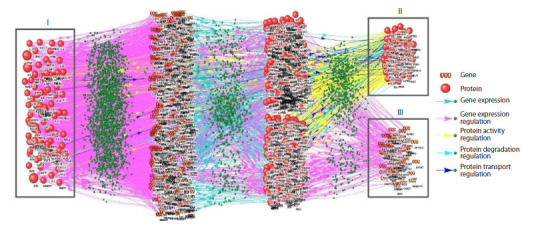
Reconstructed consolidated network of cytokine interactions with ASD-related proteins and genes Roman numerals indicate: I, cytokines regulating ASD-associated proteins and genes, II, ASD-associated proteins regulated by cytokines, III, ASD-associated genes regulated by cytokines.

Integration of the reconstructed subnetworks produced a consolidated network representing cytokine interactions with ASD-associated proteins and genes (Fig. 2). This integrated network contained 1,112 nodes classified into two distinct types and 3,675 specific interactions between them, as detailed in Table 1.

**Table 1.**
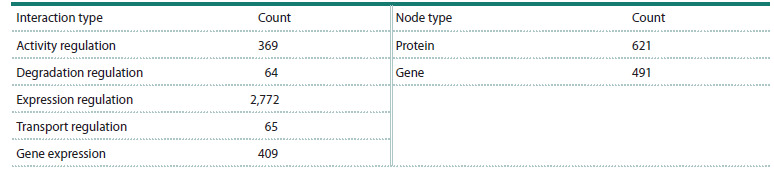
Types and quantities of nodes and interactions in the consolidated gene network of cytokine interactions with ASD-associated proteins and genes

Network analysis identified 110 regulatory cytokines (Fig. 2, I) targeting 58 ASD-associated proteins (Fig. 2, II) and 91 ASD-related genes (Fig. 2, III).

**Functional enrichment analysis of the cytokine-regulated gene set**

Gene Ontology enrichment analysis was performed using the DAVID platform on the subset of ASD-associated genes identified as being under cytokine regulatory control in the reconstructed consolidated network. This analysis revealed significant enrichment (FDR < 0.05, false discovery rate) for 56 biological processes related to nervous system development and function. Specifically, these cytokine-regulated genes were overrepresented in processes including dendritic spine morphogenesis, hippocampal development, and neuronal migration (Table 2). Only the most statistically significant and biologically specific processes are presented in Table 2, while general cellular processes such as transcriptional regulation were excluded from the final selection.

**Table 2.**
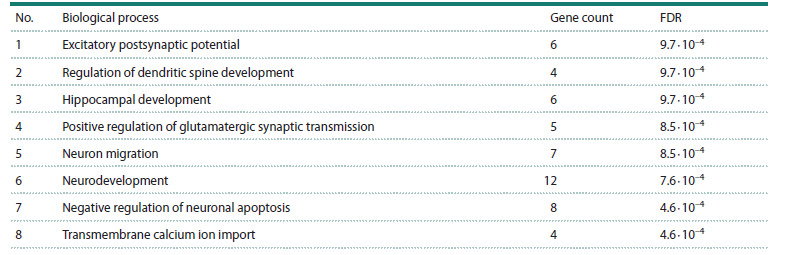
Enrichment analysis of ASD-associated genes from the integrated network that are regulated by cytokines Note. FDR, false discovery rate.

**Cytokine prioritization**

To identify cytokines with the greatest impact on the regulatory network, we conducted multi-criteria prioritization based on three network topological and functional parameters: node degree, betweenness centrality, and enrichment in ASDassociated biological processes.

To evaluate the involvement of cytokines in ASD-associated biological processes, we developed a custom script that processes two primary inputs: cytokines identified through network reconstruction, and ASD-associated biological processes derived from Gene Ontology enrichment analysis of SFARI gene sets. The algorithm assessed each cytokine’s involvement in the listed ASD-associated biological processes. This analysis identified 13 cytokines that participate in biological processes implicated in ASD (FDR <0.05, Table 3).

**Table 3.**
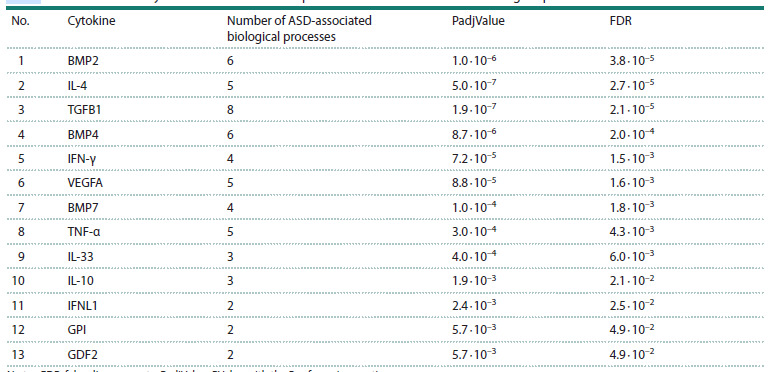
Prioritization of cytokines based on their representation in ASD-associated biological processes Note. FDR, false discovery rate, PadjValue, PValue with the Bonferroni correction.

To rank the cytokines by their influence within the network, two centrality metrics were employed: betweenness centrality and degree centrality. Betweenness centrality reflects the number of the shortest paths in a network that pass through a given node, while degree centrality is defined by the number of its direct connections to other nodes. These metrics quantify a node’s influence on the network, as higher values indicate a more significant impact of the node. 15 most influential cytokines based on each metric are presented in Table 4.

**Table 4.**
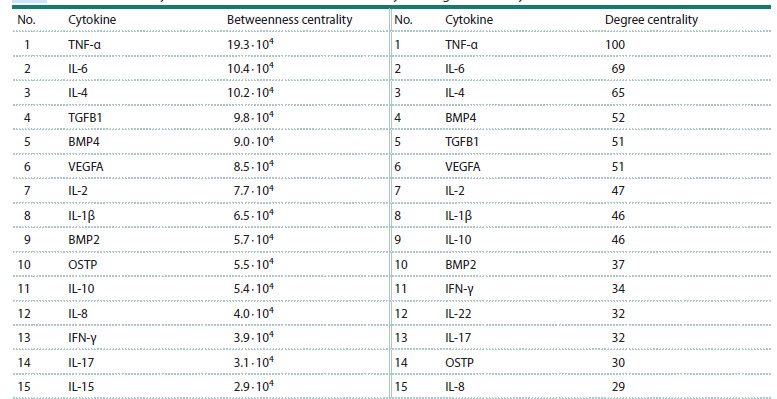
Prioritization of cytokines based on betweenness centrality and degree centrality Note. Betweenness centrality is defined as the number of the shortest paths in a network that pass through a particular node, while degree centrality represents the number of direct connections a node has with other elements in the network.

**Cytokines as potential targets for pharmacological intervention**

Based on the data presented in Tables 3 and 4, a list of 21 potentially key regulators was compiled: BMP2, BMP4, BMP7, GDF2, GPI, IFN-γ, IFNL1, IL-10, IL-33, IL-15, IL-17, IL-1β, IL-2, IL-22, IL-4, IL-6, IL-8, OSTP, TGFB1, TNF-α, and VEGFA. Validation of this list against the GETdb database confirmed the status of these cytokines as promising pharmacological targets.

According to the DrugBank database records, 8 out of the 21 cytokines (TNF-α, IL-6, IL-4, VEGFA, IL-2, IL-1β, IFN-γ, IL-17) are established targets for approved pharmaceuticals. Notably, four of these (IL-4, VEGFA, TNF-α, and IFN-γ) were also identified among the eight highest-priority candidates in our analysis, which were ranked based on a consensus across multiple prioritization metrics (IL-4, TGF-β1, BMP4, VEGFA, BMP2, IL-10, IFN-γ, and TNF-α).

In clinical practice, drugs targeting cytokines TNF-α, IL- 6, IL-4, VEGFA, IL-2, IL-1β, IFN-γ, and IL-17 are primarily used as immunosuppressants and antitumor agents. The therapeutic mechanisms of these agents principally involve either receptor blockade, utilizing cytokine antagonists, or direct cytokine neutralization through monoclonal antibodies

## Discussion

Analysis of Tables 3 and 4 identified 21 cytokines (BMP2, BMP4, BMP7, GDF2, GPI, IFN-γ, IFNL1, IL-10, IL-33, IL-15, IL-17, IL-1β, IL-2, IL-22, IL-4, IL-6, IL-8, OSTP, TGFB1, TNF-α, and VEGFA) as potential pharmacological targets, based on the GETdb database. Cross-referencing with the DrugBank database revealed that eight of them (TNF-α, IL-6, IL-4, VEGFA, IL-2, IL-1β, IFN-γ, and IL-17) are already targeted by approved therapeutics. A review of the existing literature confirms the critical role of specific proinflammatory cytokines (TNF-α, IL-6, IL-2, IL-1β, IFN-γ, VEGFA, IL-17A) in CNS development and function. These factors, secreted by classically activated microglia, are key drivers of neuroinflammation. Furthermore, dysregulation of specific cytokines, such as IL-6, IFN-γ, and IL-17A, during gestation, induced by maternal immune activation, may alter embryonic brain development and predispose to autism spectrum disorder (ASD) (Fujitani et al., 2022; Majerczyk et al., 2022).

Studies using maternal immune activation (MIA) mouse models demonstrate that CD4+ T-lymphocytes from affected offspring exhibit elevated IL-17A production (Morgan et al., 2010; Parkhurst et al., 2013). Furthermore, it was established that the activity of maternal RORγt-expressing proinflammatory T-cells (Th17), the primary source of IL-17A, is a prerequisite for the induction of ASD-like phenotypes in the offspring. It was further demonstrated that ASD-like phenotypes in the offspring require the activity of maternal RORγt-expressing Th17 cells, which are the primary source of IL-17A. Choi G.B. et al. (2016) demonstrated that both IL-17A neutralization and direct targeting of Th17 cells in pregnant mice prevent the development of MIA-induced behavioral abnormalities in their offspring. Conversely, the administration of IL-17A into the fetal brain was shown to cause disruptions in cerebral hemisphere development and the manifestation of ASD-associated symptoms. These behavioral manifestations are linked to altered right-hemispheric activity, a region critical for adaptation mechanisms (Nikolaeva, Vergunov, 2020). This lateralized dysfunction is further supported by the significantly higher prevalence of left-handedness in children with ASD (Nikolaeva, Gaidamakina, 2018).

Paradoxically, despite the documented role of IL-17A in impairing CNS development, emerging evidence indicates its therapeutic potential for normalizing behavioral deficits in adult offspring of mothers with MIA. A study by M. Reed et al. (2020) demonstrated that lipopolysaccharide (LPS) therapy normalized behavior in adult offspring from mothers with immune activation (MIA); however, it was ineffective in monogenic models of autism spectrum disorder. This divergent outcome was attributed to variations in cytokine secretion, specifically a significantly lower production of IL- 17A in response to LPS in monogenic models compared to MIA-induced counterparts

In addition to pro-inflammatory cytokines, anti-inflammatory cytokine IL-4 is involved in ASD pathogenesis. This cytokine is critical for inducing the alternative activation pathway of microglia (M2 phenotype). Microglia in the M2 state exhibit anti-inflammatory and reparative functions, which include the secretion of numerous growth factors such as IGF-I, FGF, CSF1 and neurotrophic factors (Sica, Mantovani, 2012). Subsequently, these factors activate Trk receptors, a family of receptor tyrosine kinases involved in the regulation of synaptic plasticityStudies have identified a significant elevation of IL-4 levels in the amniotic fluid and maternal serum during pregnancy in women whose children were later diagnosed with ASD (Goines, Ashwood, 2013). The role of increased IL-4 concentration in ASD pathogenesis, however, remains unclear: it could either contribute to the development of pathology or represent a compensatory mechanism in response to inflammatory processes.

We hypothesize that repurposing established clinical cytokines offers a viable path for ASD therapy. To test this, we propose to initiate studies analogous to those by M. Reed et al. (2020), utilizing agents targeting the cytokines TNF-α, IL-6, IL-4, VEGFA, IL-2, IL-1β, IFN-γ, and IL-17, with existing clinical applications. Planning of future research must account for the variable efficacy of cytokine interventions, which is influenced by disease etiology and developmental stage. A comprehensive approach should involve the use of rodent models that represent distinct methods of inducing ASD and its various forms, followed by a comparative analysis of the resulting data. This methodology will facilitate a more profound understanding of the effects of cytokines on the development and symptoms of ASD of diverse origins, as well as an assessment of the potential for repurposing the corresponding pharmaceutical agents for treating and alleviating ASD symptoms.

## Conclusion

• Using the ANDSystem knowledge base and its components, we performed a computer-based reconstruction of five specialized gene subnetworks. These subnetworks represent distinct pathways through which cytokines influence proteins and genes associated with autism spectrum disorder (ASD), thereby providing a comprehensive mapping of cytokine interactions with ASD-associated biomolecules. Through the integration of these subnetworks into a unified model, a network for cytokine regulation of ASD-associated genes and proteins was reconstructed for the first time. The consolidated network comprises 1,112 nodes of two types (491 genes and 621 proteins) interconnected by 3,675 edges representing five distinct types of interactions.

• Analysis of the final gene network enabled the identification of 110 cytokines that regulate the activity, transport, and stability of network components implicated in ASD. Furthermore, 58 proteins and 91 genes involved in ASD pathogenesis, all of which are under cytokine regulation, were identified. Key characteristics of the network were defined, providing evidence for the significant role of cytokine- mediated regulation in ASD pathogenesis, and revea- ling specific cohorts of ASD-linked genes under cytokine control.

Subsequent Gene Ontology (GO) enrichment analysis for biological processes was performed on the subset of ASDassociated genes identified as being under cytokine regulatory control in the reconstructed interaction network. This analysis revealed 56 statistically significant biological processes related to neurodevelopment. Notable among these were dendritic spine morphogenesis, hippocampal development, neuronal migration, and the regulation of synaptic transmission.

• Cytokine prioritization was conducted to pinpoint key regulators, employing an analysis of network metrics (betweenness centrality and node degree) alongside an evaluation of functional relevance via linkage to ASDassociated GO biological processes. This approach yielded a set of 21 cytokines, with 8 (IL-4, TGF-β1, BMP4, VEGFA, BMP2, IL-10, IFN-γ, TNF-α) ranking highest across all evaluated parameters.

Notably, 8 out of the 21 key cytokines (TNF-α, IL-6, IL-4, VEGFA, IL-2, IL-1β, IFN-γ, IL-17) are targeted by existing, clinically approved drugs, highlighting an opportunity for repurposing immunomodulatory agents for ASD. The other 13 cytokines are potential targets for compounds in clinical development. Further in vitro and in vivo studies are required to delineate the precise mechanisms through which these cytokines influence neurodevelopment and to assess the therapeutic efficacy of their modulation.

## Conflict of interest

The authors declare no conflict of interest.
